# Antiviral Strategies against Arthritogenic Alphaviruses

**DOI:** 10.3390/microorganisms8091365

**Published:** 2020-09-07

**Authors:** Rana Abdelnabi, Leen Delang

**Affiliations:** Department of Microbiology, Immunology and Transplantation, Rega Institute for Medical Research, Laboratory of Virology and Chemotherapy, Herestraat 49, 3000 Leuven, Belgium; rana.abdelnabi@kuleuven.be

**Keywords:** arbovirus, alphavirus, chikungunya, antivirals, capping, protease, replication

## Abstract

Alphaviruses are members of the *Togaviridae* family that are mainly transmitted by arthropods such as mosquitoes. In the last decades, several alphaviruses have re-emerged, causing outbreaks worldwide. One example is the re-emergence of chikungunya virus (CHIKV) in 2004, which caused massive epidemics in the Indian Ocean region after which the virus dramatically spread to the Americas in late 2013. Besides CHIKV, other alphaviruses, such as the Ross River virus (RRV), Mayaro virus (MAYV), and Venezuelan equine encephalitis virus (VEEV), have emerged and have become a serious public health concern in recent years. Infections with the Old World alphaviruses (e.g., CHIKV, RRV) are primarily associated with polyarthritis and myalgia that can persist for months to years. On the other hand, New World alphaviruses such as VEEV cause mainly neurological disease. Despite the worldwide (re-)emergence of these viruses, there are no antivirals or vaccines available for the treatment or prevention of infections with alphaviruses. It is therefore of utmost importance to develop antiviral strategies against these viruses. We here provided an overview of the reported antiviral strategies against arthritogenic alphaviruses. In addition, we highlighted the future perspectives for the development and the proper use of such antivirals.

## 1. Introduction

Alphaviruses are a group of human and animal viruses that belong to the family Togaviridae. Alphaviruses are mainly transmitted by the bite of hematophagous arthropods (e.g., ticks and mosquitoes) [1]. Classically, alphaviruses are divided into the New World and Old World alphaviruses based on their historical geographical abundance. New World alphaviruses cause encephalitic diseases and include the Venezuelan and Western equine encephalitis viruses (VEEV and WEEV) [2]. Infections with Old World alphaviruses, such as the chikungunya virus (CHIKV) and Ross River virus (RRV), mainly result in rheumatic disease that causes debilitating pain in the joints [2]. The symptoms of acute disease caused by these viruses include fever, bilateral symmetrical arthritis, and sometimes skin rash. Although acute infections by arthritogenic alphaviruses are self-limiting, several patients suffer from a chronic polyarthritis that can severely incapacitate the patient for weeks and even up to several years after the acute stage [3]. Recent outbreaks of arthritogenic alphaviruses such as CHIKV have also been associated with neurological manifestations, e.g., myelopathy, Guillain-Barré syndrome, and meningoencephalitis, especially in elderly patients with comorbidities and neonates [4].

Several arthritogenic alphaviruses have (re-)emerged worldwide and have become a major public health threat. However, there is no approved antiviral drug or vaccine for the treatment or prevention of these viral infections. The current treatment depends on symptomatic relief using analgesics, antipyretics, nonsteroidal anti-inflammatory drugs, and, in severe cases, methotrexate [2].

## 2. Transmission and Epidemiology

Arthritogenic alphaviruses are generally maintained in the wild via enzootic/epizootic transmission cycles between an arthropod vector (mostly mosquitoes) and animal reservoirs such as primates, forest birds, and macropods [2,5]. On certain occasions, human infections can occur by direct spillover from these cycles via the bite of infected mosquitoes [2,5]. To date, only CHIKV showed the ability to be transmitted in urban cycles, i.e., human–mosquito–human, similar to some flaviviruses [5].

Various mosquito species have shown the ability to transmit arthritogenic alphaviruses. For example, CHIKV is transmitted mainly by *Aedes* mosquitoes [2], whereas the closely related alphavirus O’nyong-nyong virus (ONNV) is transmitted by Anopheles species [6]. The primary vectors for Mayaro virus (MAYV) are mosquitoes of the Haemagogus species, e.g., *H. janthinomys* [7]. However, laboratory experiments proved that other mosquitoes, such as *Ae. aegypti*, *Ae. albopictus* [8], and some Anopheles species, are competent vectors for MAYV. In addition, the transmission cycles of RRV involve various mosquito species, such as those belonging to the genera of *Culex*, *Aedes*, *Anopheles*, and *Mansonia* [9].

The majority of arthritogenic alphaviruses are currently endemic to specific geographical regions or continent. RRV [10] and Barmah forest virus (BFV) [11] are endemic in Australia, ONNV in Sub-Saharan Africa [6], and MAYV in the South and the Central Americas [7]. In contrast, CHIKV is endemic in several continents. In 2004, CHIKV re-emerged in South East Asia and the Indian ocean islands and, from there, it spread to other countries, resulting in massive outbreaks with high morbidity rates [3]. In December 2013, local transmission of CHIKV was reported for the first time in the Americas. Since then, millions of CHIKV cases have been reported in the Caribbean region and several countries of Central and South America [3]. The change in mosquito vectors distribution, caused by global warming, virus adaptation to new mosquito species, and the increase in the international travel, could result in similar expansion of other arthritogenic alphaviruses in the future.

## 3. Alphavirus Replication Cycle

**Figure 1:**
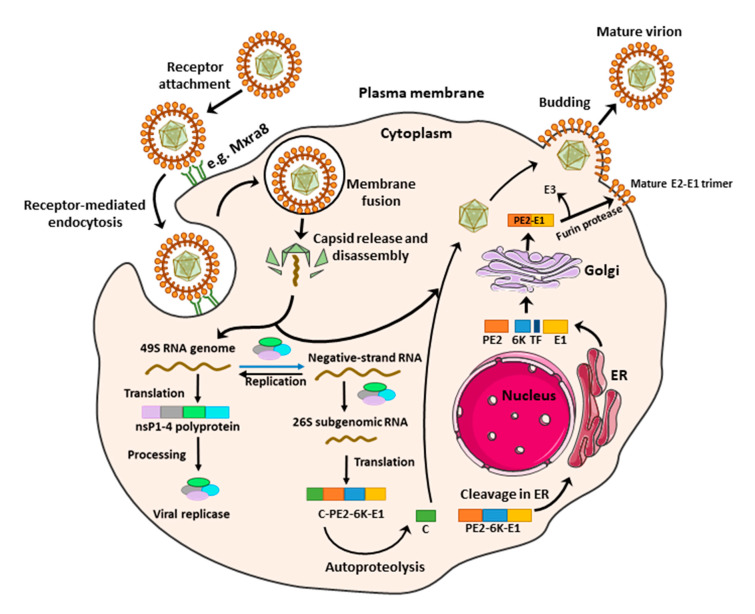
Replication cycle of arthritogenic alphaviruses. Alphaviruses enter the host cell by endocytosis following the binding of the envelope glycoprotein E2 to specific receptor(s) on the cell surface. Within the endosome, the low pH triggers the fusion of the viral envelope glycoprotein E1 with the endosomal membrane, leading to the release of the nucleocapsid into the cytoplasm. The nucleocapsid disassembles to liberate the viral genome, which is translated to produce the viral nonstructural proteins (nsP1–4). After processing, the nonstructural proteins complex to form the viral replicase, which catalyzes the synthesis of a negative-sense RNA strand to serve as a template for synthesis of both the full-length positive-sense genome and the subgenomic (26S) RNA. The subgenomic (26S) RNA is translated to produce the structural polyprotein (C-E3-E2-6K-TF-E1), which is then cleaved and processed in the Golgi apparatus and endoplasmic reticulum (ER) to produce the individual structural proteins, followed by assembly of the viral components. The assembled virus particle is released by budding through the plasma membrane, where it acquires the envelope with embedded viral glycoproteins. C = Capsid protein, E = Envelope glycoprotein, TF = Transframe protein.

Alphaviruses enter the host cell through receptor-mediated endocytosis forming an endosome. Within the endosome, conformational changes of the viral envelope glycoprotein E1 are triggered by low pH, leading to its fusion with the endosomal membrane (Figure 1) [12]. Consequently, the viral nucleocapsid is released into the cytoplasm where it disassembles to release the viral RNA genome. The 5′-proximal open reading frame of the incoming genome can be directly translated into the nonstructural (ns) polyprotein, which is processed into individual ns proteins that assemble into the viral replication complex [12]. The negative strand RNA is formed and serves as a precursor for both the positive strand genomic RNA and the subgenomic RNA, also known as 26S RNA. The 26S RNA serves as the mRNA for the generation of the viral structural proteins. The viral capsid protein is then released by its autoprotease activity, whereas the remaining polyprotein is further processed in the endoplasmic reticulum (ER) and Golgi system (Figure 1). Furin protease cleaves the pE2 into the envelope glycoproteins (E) E2 and E3 and the capsid proteins interact with genomic RNA to encapsidate the genome and form nucleocapsids. Finally, the nucleocapsids are transported to the plasma membrane and bud with the host membrane to form mature virus that is released in the environment (Figure 1) [12].

## 4. Antiviral Strategies

In theory, all steps in the alphavirus replication cycle can be potential targets for antiviral drug development. These steps involve an interplay between viral proteins and host factors. The major advantage of host-targeting antivirals is the potential broad-spectrum activity against more than one alphavirus and the lower possibility that the virus will develop resistance. However, caution must be taken to avoid (serious) side effects when targeting a host factor. We here reviewed the reported antiviral strategies against arthritogenic alphaviruses replication.

### 4.1. Virus-Targeting Inhibitors

#### 4.1.1. Early-Stage Inhibitors

To date, the entry receptors for arthritogenic alphaviruses are not fully elucidated. Interestingly, the adhesion molecule Mxra8 has been recently identified as an entry receptor for several arthritogenic alphaviruses including CHIKV, ONNV, RRV, and MAYV [13]. Treatment of different cell lines with a Mxra8–Fc fusion protein or anti-Mxra8 monoclonal antibody proved to inhibit CHIKV infection. In addition, blocking Mxra8 with both molecules reduced CHIKV and ONNV infection and disease symptoms in C57BL/6 mice [13]. 

**Figure 2:**
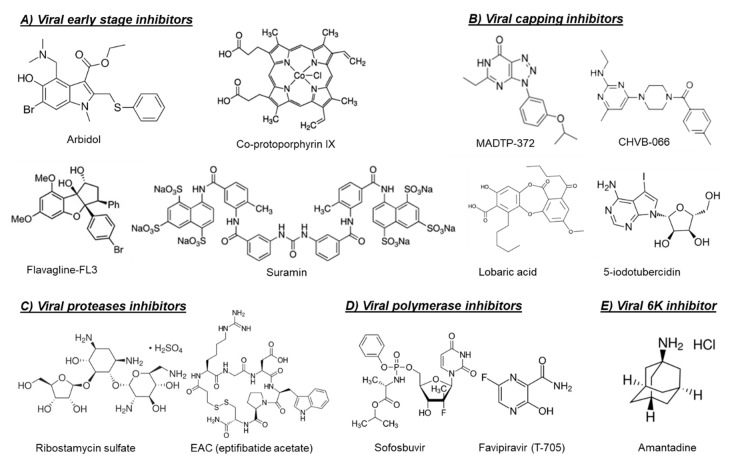
Chemical structures of selected virus-targeting alphavirus inhibitors.

Another possible target for blocking viral entry is prohibitin. Prohibitin-1 is a signaling protein that was previously identified as a receptor for CHIKV in mammalian cells [14]. Targeting prohibitin-1 with synthetic flavaglines derivatives (Figure 2) inhibited the in vitro CHIKV replication and reduced the colocalization of prohibitin-1 and the CHIKV E2 glycoprotein, suggesting an effect on CHIKV binding to this receptor [15]. 

The broad-spectrum antiviral drug arbidol (Figure 2) has also been reported as an early-stage inhibitor of CHIKV replication in vitro [16]. An arbidol-resistant CHIKV variant was identified to carry a glycine to an arginine (G407R) mutation in the viral E2 glycoprotein, which is the protein involved in the viral binding to host receptors [16]. Suramin, an anti-trypanosomiasis drug (Figure 2), has also been reported to inhibit the replication of different CHIKV isolates and related alphaviruses in vitro and in vivo [17,18,19]. Suramin proved to interact directly with the viral particles of SFV and CHIKV and hence prevented viral attachment to the host cells [20]. Moreover, suramin showed the ability to interfere with the conformational changes of viral envelope glycoproteins required for the fusion step [20].

#### 4.1.2. Viral Capping Inhibitors

Viral mRNA capping plays an essential role in subsequent downstream processing, translation, nuclear export, and stability of mRNA. For alphaviruses, the viral RNA capping involves the RNA methyltransferase (MTase) and guanylyltransferase (GTase) activities of nsP1, as well as the RNA triphosphatase function of nsP2. Interestingly, the alphavirus capping mechanism is unique and proceeds in a sequence that is distinct from the capping mechanism in the host cell [21]. Therefore, alphavirus RNA capping could be an attractive target for drug development.

The first class of small molecules reported to target nsP1 is the MADTP series, which has a triazolopyrimidinone scaffold [22,23] (Figure 2). This class of compounds showed activity against different clinical CHIKV isolates in vitro and inhibited the activity of VEEV nsP1 in an enzymatic assay [22,23]. A MADTP-resistant CHIKV variant was selected in cell culture that carried a P34S mutation in nsP1 [23]. Recently, another class of CHIKV nsP1-targeting compounds, i.e., the CHVB series, was described (Figure 2) [24,25]. In enzymatic assays, CHVB proved a potent inhibitor of the MTase and GTase activities of nsP1 of Semliki Forest virus (SFV) and VEEV [25]. A CHVB-resistant virus proved cross-resistant to the MADTP series, suggesting a similar mode of action. However, in contrast to the MADTP series, for which only one amino acid substitution was enough to develop full resistance, resistance to CHVB compounds required the presence of at least two mutations in nsP1 (i.e., S454G and W456R) [25]. A high throughput ELISA assay resulted in the identification of 18 additional compounds that inhibited the GTase activity of VEEV nsP1 [26]. Interestingly, two compounds in the tested series could still inhibit the GTase activity of VEEV nsP1 carrying the MADTP resistance mutation (D34S), suggesting that these compounds have a different mechanism of action [26].

In another study, a fluorescence polarization-based GTP competition screen led to the identification of several hit compounds that were able to compete with GTP for the CHIKV nsP1-GTP binding site [27]. One of these compounds was the naturally derived compound lobaric acid (Figure 2) and its antiviral activity against SINV and CHIKV was also confirmed in cell-based antiviral assays [27]. More recently, 6′-β-fluoro-homoaristeromycin and 6′-fluoro-homoneplanocin A have been reported as potent inhibitors of CHIKV replication [28]. Both compounds proved to target the MTase activity of SFV nsP1 in enzymatic assays [28]. The adenosine analog 5-iodotubercidin (5-IT, Figure 2) has also been recently reported to inhibit the MTase activity of CHIKV nsP1 in a capillary electrophoresis-based enzymatic assay and to potently reduce CHIKV replication in cell culture [29].

#### 4.1.3. Viral Protease Inhibitors

The viral protease has been shown to be an excellent target to inhibit replication of human immunodeficiency virus (HIV) and hepatitis C virus (HCV) [30]. Similarly, the alphavirus nsP2 and capsid protease activities could be promising targets for drug discovery and development. The cysteine protease activity of the alphavirus nsP2 is located in its C-terminal region and is needed for processing of the nonstructural viral polyprotein [31]. On the other hand, the C-terminal part of the capsid protein (CP) possesses a serine auto-protease activity, which is essential for its release from the structural polyprotein [32].

The crystal structures or homology models of the nsP2 protease of different arthritogenic alphaviruses were extensively explored in molecular docking and molecular dynamics studies in order to identify potential alphavirus inhibitors [33,34,35,36,37]. In one of these studies, some hit compounds and five potential binding pockets of the CHIKV nsP2 protease were identified, which may help to design potent CHIKV protease inhibitors in the future [33]. Furthermore, five arylalkylidene derivatives of 1,3-thiazolidin-4-one were reported to inhibit the in vitro CHIKV replication with EC_50_ values in the low µM range. Based on molecular docking studies, it was proposed that these compounds may interact with the nsP2 protease domain [36]. However, for most of the predicted compounds in such studies, there was no direct experimental evidence that these compounds inhibit the protease activity. Interestingly, a panel of in silico predicted CHIKV protease inhibitors were confirmed to inhibit the enzymatic activity in CHIKV nsP2 cell-free protease assays, as well as to inhibit the in vitro replication of CHIKV [35]. Recently, a library of Food and Drug Administration (FDA)-approved drugs and known cysteine protease inhibitors were docked into the crystal structure of CHIKV nsP2 protease as an attempt for drug repurposing [37]. Computational analysis and molecular dynamics simulations led to the identification of ribostamycin sulfate (an aminoglycoside-aminocyclitol antibiotic, Figure 2) as a potential inhibitor of CHIKV nsP2 protease [37]. However, the ability of this compound to inhibit CHIKV replication in cell culture has not been confirmed yet.

In contrast to the multiple studies targeting the nsP2 protease, studies exploring the capsid protease are scarce. The first study confirming that the alphavirus CP protease could be a potential druggable target was published very recently [32]. Three inhibitors of CHIKV CP autoprotease activity (i.e., EAC (Figure 2), AP4, and PSU) were identified by structure-based virtual screening of the LOPAC^®^ compounds library (Sigma Aldrich) against the crystal structure of CHIKV CP protease [32]. The anti-CP proteolytic activity of these compounds were confirmed in a FRET-based proteolytic assay and they also proved to inhibit CHIKV replication in Vero cells [32].

#### 4.1.4. Viral RNA-Dependent-RNA Polymerase Inhibitors

Antiviral drugs that target the viral polymerase are currently available for treatment of the infections with several viruses including HIV, hepatitis B virus (HBV), HCV, and herpes. For alphaviruses, the viral nsP4 protein possesses the viral RNA-dependent RNA polymerase (RdRp) activity and could be a potential target for development of broad-alphavirus inhibitors [31]. Favipiravir (also named T-705, Figure 2) is a broad-spectrum antiviral that has been approved in Japan for treatment of pandemic influenza virus [38]. Favipiravir and its defluorinated analog, T-1105, have been shown to inhibit the in vitro replication of CHIKV and related (arthritogenic) alphaviruses [39]. Treatment of CHIKV-infected AG129 mice with favipiravir (300 mg/kg/day for 7 days) prevented the development of severe neurological disease and markedly increased the survival rate [39]. In C57BL/6J mice, favipiravir treatment (300 mg/kg/day for 4 days) also reduced viral replication in the joints of the extremities during the acute phase of infection and prevented systemic viral spread [40].

The approved HCV polymerase inhibitor sofosbuvir, a nucleotide analog (Figure 2), has been shown to inhibit the replication of CHIKV efficiently in Huh-7 cells and, to a lesser extent, in iPSC-derived human astrocytes [41]. Sofosbuvir (20 mg/kg/day) protected against CHIKV-induced disease in adult Swiss mice and increased survival in neonates when administered at 40 mg/kg/day and 80 mg/kg/day doses [41].

#### 4.1.5. Viral 6K Inhibitors

The alphavirus 6K protein is considered a viroporin due to its ability to form ion channels that facilitate viral assembly and release through the cellular membranes [42]. Recently, the CHIKV 6K protein was functionally characterized using a combination of electrophysiology, confocal, and electron microscopy, as well as molecular dynamics [43]. In the same study, the influenza M2 ion channel inhibitor amantadine (Figure 2) proved to inhibit the ion channel forming ability of CHIKV 6K protein and to alter the morphology of CHIKV virus-like particles (VLP) [43]. Moreover, amantadine reduced CHIKV viral titers in cell culture, suggesting that the alphavirus 6K protein could be a promising druggable target [43].

#### 4.1.6. Virucidal Compounds

Several natural products have been reported to exert virucidal activity against arthritogenic alphaviruses [44,45,46]. Such compounds directly interact with the viral particle, leading to virus inactivation. The phospholipases A2 (PLA2s) from snake (*Crotalus durissus terrificus*) venom have been reported to have a virucidal effect against several enveloped viruses, including CHIKV [44]. Another example is ginkgolic acid, isolated from the *Ginkgo biloba* plant, which has been recently shown to inhibit the in vitro replication of CHIKV and MAYV through a direct virucidal effect [45]. Dolastane (isolated from Seaweed *Canistrocarpus cervicornis*) also had potent virucidal activity against CHIKV [46]. The use of amphipathic molecules, such as Co-protoporphyrin IX (Figure 2), to disrupt the alphavirus envelope structure has also been shown to block the entry of CHIKV, MAYV, and Sindbis virus (SINV) in cells [47].

### 4.2. Host-Targeting Inhibitors

#### 4.2.1. Endosomal Fusion Inhibitors

**Figure 3:**
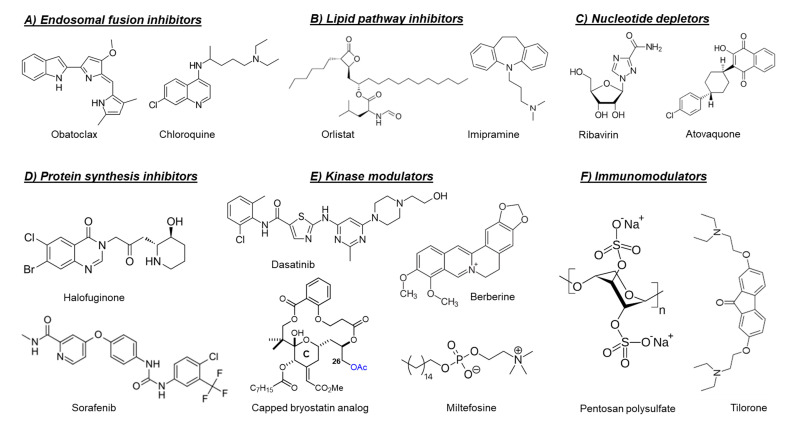
Chemical structures of selected host-targeting alphavirus inhibitors.

As mentioned before, the fusion of the viral envelope glycoprotein E1 with the endosomal membrane is triggered by low pH in the endosome [12]. Consequently, compounds that raise the endosomal pH, such as chloroquine [48,49] and obatoclax (Figure 3) [50], have been reported to inhibit the in vitro replication of arthritogenic alphaviruses, e.g., CHIKV and SFV by preventing the viral fusion step. However, a clinical trial of chloroquine in CHIKV-infected patients during the acute phase of infection did not show a significant efficacy over meloxicam (a nonsteroidal anti-inflammatory drug) [51].

#### 4.2.2. Lipid Pathways Inhibitors

The envelope of alphavirus particles is acquired during budding from the lipid plasma membrane and plays an important role during virus entry into the host cell. The lipid composition of the alphavirus envelope has been demonstrated to be critical for virus particle stability and infectivity [52]. It has also been reported that the presence of sphingolipids and cholesterol in the target host membranes is essential for alphavirus envelope fusion and for viral exit [53]. Therefore, targeting the host lipid pathways could be a promising strategy for inhibition of arthritogenic alphaviruses.

The fatty acid synthase (FASN) and stearoyl-CoA desaturase (SCD1) are two key enzymes which are essential for the *de novo* synthesis of long-chain fatty acids and their early desaturation, respectively [54]. Both enzymes were shown to play an important role during the replication of arthritogenic alphaviruses such as CHIKV and MAYV [54,55]. Inhibition of FASN activity (by the anti-obesity drug orlistat (Figure 3) [54,55] or the antibiotic cerulenin [54]), as well as the SCD1 activity (by CAY10566) [54], reduced the in vitro replication of both CHIKV and MAYV. Interference of the cellular cholesterol trafficking by the antidepressant drug imipramine (Figure 3) reduced CHIKV replication in human skin fibroblasts in a dose-dependent manner [56].

Other potential targets in the host lipid pathway are the liver X receptors (LXRα and LXRβ), host transcription factors essential for the intracellular cholesterol homeostasis through regulation of cholesterol exporters [57]. The LXRβ-selective agonist, LXR-623, has been shown to inhibit CHIKV replication in human fibroblasts in a dose-dependent manner [57]. This antiviral effect was partially reversed by cotreating the cells with exogenous cholesterol [57].

#### 4.2.3. Protein Synthesis Inhibitors

Halofuginone (Figure 3) is a potent inhibitor of the prolyl tRNA synthetase enzyme that results in the accumulation of uncharged prolyl tRNAs and thus forces the cell to shut down the translation [58]. Interestingly, halofuginone suppressed CHIKV and ONNV replication in human skin fibroblasts through inhibition of the viral protein synthesis [59]. The anticancer drug sorafenib (Figure 3) also showed antiviral activity against CHIKV and SINV in vitro [60]. The inhibition of viral translation by sorafenib was suggested to be due to dephosphorylation of the eukaryotic translation initiation factor 4E (eIF4E) [60]. Silvestrol is a specific inhibitor of the RNA helicase eIF4A that inhibits mRNA translation by blocking the unwinding of RNA secondary structures in the 5′-untranslated regions (5′-UTRs) [61]. Treating CHIKV-infected cells with silvestrol inhibited viral replication and protein synthesis in the nanomolar range [61]. SR9009, on the other hand, is an agonist of the Rev-erb receptors α/β, which are transcriptional repressors belonging to the family of nuclear receptors [62]. Treating Huh-7 cells with SR9009 inhibited the replication of CHIKV and ONNV mainly at the level of subgenomic RNA translation [62]. Harringtonine, which has been previously shown to inhibit eukaryotic protein synthesis, reduced the levels of CHIKV nsP3 and E2 proteins in a dose-dependent manner [63]. This antiviral effect was also observed in primary human skeletal myoblasts, an in vivo target of CHIKV infection. Harringtonine also reduced SINV replication, suggesting that the antiviral activity may extend to other alphaviruses [63].

The ubiquitin-proteasome system plays a major role in the degradation of cellular protein and has been previously reported to regulate the intracellular levels of certain viral proteins including the alphavirus nsP4 protein [64]. The clinically approved proteasome inhibitor bortezomib has been reported to inhibit the in vitro replication of different CHIKV strains [65]. Evaluation of CHIKV protein levels after treatment showed marked reduction (up to 80%) in the structural protein levels, whereas the nsP4 protein level was strongly elevated [65]. Similarly, treating MAYV-infected cells with the proteasome inhibitors MG132 and lactacystin reduced viral titers in a dose-dependent manner and diminished the levels of E1 and nsP1 proteins [66].

#### 4.2.4. Nucleotide Depleting Compounds

The inosine monophosphate dehydrogenase enzyme (IMPDH) catalyzes an essential step in the *de novo* biosynthesis of guanine nucleotides [67]. Inhibition of IMPDH by the broad-spectrum antiviral ribavirin (a guanosine analogue, Figure 3) or the immunosuppressant mycophenolic acid reduced CHIKV replication through depletion of the intracellular GTP pool [67,68]. Another intracellular nucleotide-depleting compound is the uridine analog 6-azauridine. This compound acts as a competitive inhibitor of the orotidine monophosphate decarboxylase enzyme (OMP), resulting in depletion of the intracellular UTP pools. Consequently, 6-azauridine inhibits the replication of several viruses, including CHIKV and SFV [69]. The antiparasitic drug atovaquone (Figure 3) has also been shown to inhibit the replication of CHIKV in a dose-dependent manner [70]. The observed antiviral activity of atovaquone is suggested to be due to the inhibition of *de novo* pyrimidine biosynthesis [70].

#### 4.2.5. Cellular Kinase Modulators

Targeting cellular kinases has been reported as a potential strategy to inhibit the replication of arthritogenic alphaviruses. The Src family kinases (SFKs) comprise a group of membrane-associated kinases that mediate signal transduction of certain cellular receptors and have been reported to promote the replication of several viruses, including HCV and dengue virus (DENV) [71]. A kinome profiling study following infection of human dermal fibroblasts with CHIKV identified alterations in host kinases caused by the viral infection [71]. The kinome profile showed that the SFK-phosphatidylinositol 3-kinase (PI3K)-AKT-mTORC signaling pathway was markedly activated in CHIKV-infected cells. Both dasatinib (an SFK inhibitor, Figure 3) and Torin 1 (an mTORC1/2 inhibitor) were able to reduce the virus yield of CHIKV and of other alphaviruses, e.g., ONNV, RRV, and MAYV, in human fibroblasts [71]. Dasatinib reduced the accumulation of CHIKV structural proteins with no effect on viral RNA replication [71]. This effect was attributed to the inhibition of alphavirus subgenomic RNA translation [71]. Inhibition of AKT-phosphorylation by miltefosine (anti-leishmaniosis drug, Figure 3) also decreased CHIKV replication in human dermal fibroblasts [72].

Another example of involved cellular kinase pathways is the major mitogen-activated protein kinase (MAPK) signaling pathway. This pathway was found to be activated during CHIKV infection and to play a role in infectious alphavirus particles formation [73]. Reducing the MAPK pathway activation by the plant-derived alkaloid berberine (Figure 3) resulted in inhibition of ONNV and CHIKV replication in cell culture. Treatment of CHIKV-infected C57BL/6 mice with berberine (10 mg/kg/daily from day 1 to 6 post-infection) reduced viremia and disease symptoms in mice [73]. Recently, berberine has been shown to interfere with the alphavirus nucleocapsid assembly and hence inhibit the formation of infectious virus particles [74].

A different example is the protein kinase C (PKC) family which comprises related serine/threonine kinases that regulate many cellular processes such as proliferation, differentiation and apoptosis [75]. PKC activators, such as the phorbol ester prostratin [75] or salicylate-based bryostatin analogs [76], have been shown to inhibit the replication of CHIKV in cell culture. Unlike phorbol esters, bryostatin has no tumor-promoting effect, which make bryostatin-based analogs safer and more promising for further drug development. Interestingly, capping the hydroxyl group at position C26, which is crucial for binding to PKCs in the scaffold of these bryostatin analogs (Figure 3), did not abrogate the antiviral activity [76]. The combination with different PKC inhibitors counteracted the antiviral activity of a noncapped analog but did not affect that of capped analogs, supporting that the capped analogs inhibited CHIKV replication via a PKC-independent pathway [76].

#### 4.2.6. Cellular Chloride Channels Inhibitors

Cellular ion channels have been shown be involved in the entry and genome replication of several viruses [77]. Using siRNA silencing, the cellular chloride channels CLIC1 and CLIC4 have been identified to be pro-viral factors that are required for efficient CHIKV replication [78]. The chloride channel inhibitors diisothiocyanostilbene-2,20-disulfonic acid (DIDS), 9-anthracene carboxylic acid (9-ACA), and 5-nitro-2-3-phenylpropylamino benzoic acid (NPPB) significantly reduced CHIKV progeny in human (Huh-7) cells [78]. Moreover, NPPB also reduced CHIKV titer in mosquito (C6/36) cells [78]. Mechanistic studies suggested that these chloride channels are involved in post-entry steps in the CHIKV replication cycle [78].

#### 4.2.7. Cellular Furin Inhibitors

Post-translational modifications of alphavirus structural proteins are essential for the production of mature alphavirus virions [79]. Cellular furins are involved in such modifications through processing of the viral glycoprotein precursor PE2-E1 to produce mature viral glycoproteins (Figure 1) [79]. Inhibition of cellular furins by decanoyl-RVKR-chloromethyl ketone (dec-RVKR-cmk) has been shown to reduce CHIKV infection and viral spreading in human muscle satellite cells [80].

#### 4.2.8. Sodium-Potassium ATPase Inhibitors

Digoxin is a cardiac glycoside that inhibits the sodium-potassium ATPase activity. In a high- throughput antiviral screening, digoxin has been identified as a potent CHIKV inhibitor in human cell lines [81]. Moreover, digoxin also inhibited the replication of other related alphaviruses, including RRV [81]. Mechanistic studies revealed that digoxin exerted its antiviral activity at a post-entry stage of the CHIKV replication cycle and that this effect could be reversed by addition of extracellular potassium ions [81]. Digoxin-resistant CHIKV variants were selected which carried several mutations in the viral nonstructural proteins with nsP4 V209I as key mutation, suggesting that digoxin targets the viral replicase machinery [81].

#### 4.2.9. Serotonin Receptors Modulators

Serotonin or 5-hydroxytryptamine (5-HT) receptors are mostly G-protein coupled receptors that play a key role in several physiological functions and signaling pathways [82]. Interestingly, the 5-HT receptor agonist 5-nonyloxytryptamine (5-NT) has been reported to block CHIKV infection [82]. More recently, the 5-HT antagonist methiothepin mesylate (MM) has also been shown to markedly inhibit CHIKV infection in vitro [83]. Mechanistic studies suggested that 5-NT inhibits the uncoating step, whereas MM mainly interferes with the internalization and membrane hemifusion steps of the viral replication cycle [83].

#### 4.2.10. Immunomodulators

The host innate immune responses, mainly type I interferon (IFN) signaling, play an essential role in controlling acute alphavirus infections [84]. Recombinant IFN-α has been reported to inhibit the replication of CHIKV and SFV in vitro [69]. Treating CHIKV-infected cells with a combination of ribavirin and IFN-α resulted in synergistic antiviral effect [85]. Moreover, a mathematical model predicted that a 99% reduction in CHIKV levels could be achieved by combining ribavirin and IFN-α at standard clinical regimens [85]. The IFN-inducible protein viperin has been also reported to exert antiviral effects during the in vivo replication of SINV [86] and CHIKV [87]. Notably, Viperin^−/−^ C57BL/6 mice infected with CHIKV showed more severe disease symptoms compared with the infected wild-type mice, which was attributed to an altered CD4 T-cell response [88]. Tilorone (Figure 3) is an orally available IFN-inducer that has been reported 50 years ago to have antiviral activity against SFV in mice [89]. Recently, tilorone has been also reported to inhibit CHIKV-induced CPE in Vero cells [90]. Polyinosinic acid:polycytidylic acid (poly(I:C)) is a synthetic analogue of dsRNA that has been reported to inhibit CHIKV replication both in vitro and in vivo [91,92]. The antiviral effect of poly (I:C) is mediated by upregulation of the toll-like receptor-3 (TLR3), which, in turn, results in the induction of IFN-α/β and other antiviral genes (e.g., OAS and MxA) [91,92]. The retinoic acid inducible gene-I (RIG-I) is a member of the RIG-I like receptor family, which, upon activation by viral nucleic acids, results in downstream stimulation of multiple antiviral factors [93]. Consequently, the use of synthetic dsRNA molecules as RIG-I agonists inhibited the in vitro and in vivo replication of different viruses including alphaviruses [94,95]. Recently, a novel small molecule (i.e., C11) has been identified as a potent inhibitor of the replication of alphaviruses, including CHIKV, ONNV, RRV, and MAYV [96]. This molecule acts as an agonist of the adaptor protein STING, leading to the induction of type I IFN response [96].

Another promising class of immunomodulators are the heparan sulfate mimetics (e.g., pentosan polysulfate and PG545, also called pixatimod). Heparan sulfate mimetics inhibit the glycosidase heparinase which degrades degrade heparan sulfate, a key component of the extracellular matrix. Pentosan polysulfate is used for the treatment of interstitial cystitis and osteoarthritis, whereas pixatimod is currently developed as an anticancer drug. Both drugs have been reported to reduce the severity of alphavirus-induced disease in vivo through the modulation of inflammatory infiltrates and cytokine levels [97,98]. Treatment of mice infected with RRV or CHIKV with pentosan polysulfate (Figure 3) resulted in a significant disease amelioration and protection of cartilages [97]. Pentosan polysulfate is currently evaluated in phase II clinical trials for patients diagnosed with RRV-induced arthritic disease (PARA_004, Paradigm BioPharmaceuticals).

## 5. Perspectives

The worldwide re-emergence of arthritogenic alphaviruses and the high morbidity rate associated with their infections underline the need for potent and safe antiviral drugs against these viruses. Efficacious antiviral drugs, if administered in time, could reduce the severity of the disease symptoms during the acute infection by lowering the viral loads in the infected patient. Moreover, reducing viremia in infected patients using antivirals may indirectly limit virus transmission by mosquitoes and hence might reduce the chance for massive epidemics [99]. Since the severity of symptoms during the acute phase of infection could increase the chance for alphavirus-induced chronic polyarthritis [100], the use of antivirals during the acute infection may decrease the likelihood to develop chronic symptoms.

Currently, approved antiviral drugs are available for the treatment of only a limited number of viruses such as HIV, HBV, HCV, influenza, and herpes viruses. By investing sufficient time and efforts, it could also be possible to develop safe and potent antivirals for the treatment and/or prophylaxis of arthritogenic alphavirus infections. As discussed in this review, several molecules with in vitro anti-alphavirus activity have been reported, but most of these are still in the early stages of preclinical development. A next important and indispensable step is the evaluation of in vivo infection models. Only few of the reported molecules have been tested in alphavirus infection models so far. Infection models in small animals are available for several alphaviruses [1], but the existing models have limitations since they do not recapitulate all the key aspects of alphavirus disease in humans.

The development of specific antiviral drugs against each arthritogenic alphavirus separately will not be economically viable. Therefore, pan-alphavirus inhibitors will be the prime strategy to cope with this challenge. Since the alphavirus nsP2 (protease) and nsP4 (viral polymerase) proteins have conserved catalytic domains, both are considered potential targets for development of broad-spectrum antivirals for arthritogenic alphaviruses. Another promising target is Mxra8, as it functions as an entry receptor for several arthritogenic alphaviruses [13]. Designing molecules that can specifically block this receptor may therefore be a broad-spectrum strategy to control infections with various arthritogenic alphaviruses. Besides virus-specific antivirals that target a conserved alphavirus target, host-targeting antivirals have the potential to be broad-spectrum as well. The heparan sulfate mimetic pentosan polysulfate for example has already advanced to phase II clinical studies for RRV and could be considered for evaluation as a treatment for other arthritogenic alphaviruses.

Another beneficial strategy to control emerging viral infections is the repurposing of drugs that have been approved for other diseases. Because repurposed drugs have already been intensively studied in patients and thus the safety profile is well known, the clinical evaluation of such drugs could be fast-tracked during viral epidemics. Furthermore, production strategies for these drugs have been implemented. However, repurposed drugs cannot be expected to be highly potent inhibitors of alphaviruses, as these were not developed specifically against this particular virus. Examples of approved drugs that would be of interest to evaluate in clinical trials during epidemics of arthritogenic alphaviruses include sofosbuvir, favipiravir, orlistat, and tilorone, since these drugs showed promising antiviral activities, not only in cell culture, but also in vivo animal models.

Another challenge for alphavirus antiviral drug development is how such an antiviral drug could be used practically. The viremia of alphaviruses is short (<1 week). Therefore, antiviral therapy that targets virus replication should be initiated soon after the start of the infection to be efficacious. This requires an early diagnosis, but this is currently difficult. The therapeutic use of antiviral drugs could therefore be complicated. Pre-exposure prophylaxis of household members of an infected patient has been proposed as a useful strategy, since the probability of arthritogenic alphavirus transmission such as CHIKV has been reported to be up to 12% between household members [101]. Another potential application of antivirals against arthritogenic alphaviruses is prophylaxis of travelers before visiting an endemic area of a certain alphavirus. To be used as a prophylactic, an antiviral drug must be very safe and preferentially without any side effects. Prophylactic use of anti-alphavirus drugs might thus be challenging as well. To cope with the health burden and threat of (re-)emerging arthritogenic alphaviruses, the development of pan-alphavirus inhibitors will be important. More research is required to validate different approaches to obtain such pan-alphavirus inhibitors. Furthermore, a better understanding of the alphavirus life cycle and of alphavirus-induced disease is essential to provide insights that will aid to the development of broad-spectrum antiviral strategies for (arthritogenic) alphaviruses.
